# Identification of Circulating miRNAs in a Mouse Model of Nerve Allograft Transplantation under FK506 Immunosuppression by Illumina Small RNA Deep Sequencing

**DOI:** 10.1155/2015/863192

**Published:** 2015-09-08

**Authors:** Shao-Chun Wu, Cheng-Shyuan Rau, Johnson Chia-Shen Yang, Tsu-Hsiang Lu, Yi-Chan Wu, Yi-Chun Chen, Siou-Ling Tzeng, Chia-Jung Wu, Chia-Wei Lin, Ching-Hua Hsieh

**Affiliations:** ^1^Department of Anesthesiology, Kaohsiung Chang Gung Memorial Hospital and Chang Gung University College of Medicine, No. 123, Ta-Pei Road, Niao-Song District, Kaohsiung 833, Taiwan; ^2^Department of Neurosurgery, Kaohsiung Chang Gung Memorial Hospital and Chang Gung University College of Medicine, No. 123, Ta-Pei Road, Niao-Song District, Kaohsiung 833, Taiwan; ^3^Department of Plastic and Reconstructive Surgery, Kaohsiung Chang Gung Memorial Hospital and Chang Gung University College of Medicine, No. 123, Ta-Pei Road, Niao-Song District, Kaohsiung 833, Taiwan

## Abstract

*Background*. This study aimed to establish the expression profile of circulating microRNAs (miRNAs) during nerve allotransplantation in the presence and absence of FK506 immunosuppression. *Methods*. A 1 cm BALB/c donor sciatic nerve graft was transplanted into the sciatic nerve gaps created in recipient C57BL/6 mice with or without daily FK506 immunosuppression [1 mg/(kg·d)]. At 3, 7, and 14 d after nerve allotransplantation, serum samples were collected for miRNA expression analysis by Illumina small RNA deep sequencing. *Results*. Sequence analysis showed that the dominant size of circulating small RNAs after nerve allotransplantation was 22 nucleotides, followed by 23-nucleotide sequences. Nine upregulated circulating miRNAs (let-7e-5p, miR-101a-3p, miR-151-5p, miR-181a-5p, miR-204-5p, miR-340-5p, miR-381-3p, miR-411-5p, miR-9-5p, and miR-219-2-3p) were identified at 3 d, but none was identified at 7 or 14 d. Among them, miR-9-5p had the highest fold-change of >50-fold, followed by miR-340-5p with 38.8-fold. The presence of these nine miRNAs was not significant at 7 and 14 d after nerve allotransplantation with or without immunosuppression, showing that these miRNAs are not ideal biomarkers for monitoring rejection of deep-buried nerve allografts, a response usually observed later. *Conclusions*. We identified nine upregulated circulating miRNAs, which may have a biological function, particularly during the early stages after nerve allotransplantation under FK506 immunosuppression.

## 1. Background

At present, nerve allograft transplantation is a clinical reality for select patients who sustain considerable nerve injury with nerve defect [1]. In the absence of host immunosuppression, rejection of the nerve allograft typically occurs within 7 d of transplantation [2]. An immunosuppressive agent, FK506 (tacrolimus), a macrolide antibiotic derived from* Streptomyces tsukubaensis*, is used to suppress immunological responses and demonstrates relatively good results without significant rejection when applied to nerve allografts [1]. Following 3 d of pretreatment, FK506 immunosuppression (1 mg/(kg·d)) is recommended until the nerve growth has crossed the distal graft site [3]. Importantly, this compound enhances neuroregenerative effects; therefore, it shortens the required duration of immunosuppression [4–6]. However, although adequate immunosuppression after nerve allotransplantation is important for successful nerve regeneration through the allograft, the detailed mechanism underlying the role of FK506 in the immunosuppression of the nerve allograft and promotion of nerve regeneration is poorly understood [7–9]. Lack of noninvasive biomarkers of rejection may also preclude the optimization of therapeutic intervention.

Micro-RNAs (miRNAs) are endogenous, small (comprising ~ 22  nucleotides  (nt)), single-stranded, and noncoding RNAs that regulate gene expression and are detectable in whole blood, serum, plasma, urine, and other body fluids in a highly stable form [10–12]. Increasing evidence indicates that miRNAs play important roles in the posttranscriptional regulation of gene expression and are involved in the control of multiple biological and metabolic processes [13]. Circulating miRNAs present a potential technological advantage because of their remarkable stability as well as possible noninvasive and rapid diagnosis. Recently, RNA deep sequencing using next-generation sequencing has been shown to reveal a snapshot of RNA presence and quantity in a genome at a given moment in time [14]. The approach with RNA-seq is highly suited for small RNA discovery, which not only provides sequences of low abundance species but also provides quantitative data as the frequency of sequencing reads reflects the abundance of miRNAs in the population [15–17]. To date, little is known about the expression and involvement of circulating miRNAs in nerve allograft transplantation. This study aimed to establish the expression profile of circulating miRNAs with Illumina small RNA deep sequencing during nerve allograft transplantation in the presence or absence of immunosuppression.

## 2. Materials and Methods

### 2.1. Experimental Design

Male BALB/c and C57BL/6 mice (age, 10–12 weeks; weight, 30–35 g) were purchased from BioLasco (Yi-Lan, Taiwan). The BALB/c mice served as sciatic nerve allograft donors. The C57BL/6 mice were randomized into two groups of nerve allograft transplantation: one with FK506 treatment and another without FK506 treatment. These species of mice were selected on the basis of disparity at the MHC locus and prior experience with reciprocal rejection of grafts between these murine strains [18]. The mice were anesthetized by intraperitoneal injection of an anesthetic cocktail consisting of 0.1 mg/g ketamine and 0.01 mg/g xylazine. The anesthetized mice were restrained in a supine position on a heated pad to maintain the body temperature at 37°C. Under aseptic conditions, with sterile povidone/iodine preparation and 70% ethanol and sterile instruments and drapes, the skin over the proximal right hindlimb was incised, and the underlying biceps femoris muscle was bluntly dissected to expose the sciatic nerve. An established mouse sciatic nerve allotransplantation model was used. In brief, 1 cm BALB/c donor sciatic nerve grafts were transplanted in reverse orientation into the 0.5-cm sciatic nerve gaps created in the recipient BALB/c mice. Tension-free repair was then performed under an operating microscope with three 11–0 nylon (Ethicon Inc., Somerville, NJ) interrupted epineurial sutures, under 40x magnification. The muscle was closed with 5–0 vicryl sutures, and the skin was closed, with interrupted 5–0 nylon sutures. The animals were monitored to ensure appropriate feeding and diet after surgery. FK506 (1 mg/(kg·d)) was administered subcutaneously throughout the experimental course. At 3, 7, and 14 d after initial surgery (*n* = 3 animals/group at each time point), whole blood samples were collected at the indicated times in tubes containing anticoagulant. The labels (F)3d, (F)7d, and (F)14d were used to indicate the serum samples of mice undergoing allotransplantation with FK506 immunosuppression for 3, 7, and 14 d, respectively, while the labels (N)3d, (N)7d, and (N)14d were used to indicate the serum samples of mice undergoing allotransplantation without FK506 immunosuppression and sacrificed at postoperative days 3, 7, and 14, respectively. After the whole blood samples were incubated at room temperature for 15 min, they were centrifuged at 3,000 ×g for 10 min, white blood cells were slowly removed from the corresponding layers, and the serum was extracted and stored at –80°C before processing for RNA analyses. All housing conditions and surgical procedures, analgesia, and assessments were in accordance with national and institutional guidelines; an Association for Assessment and Accreditation of Laboratory Animal Care- (AAALAC-) accredited SPF facility was used. Animal protocols were approved by the Institutional Animal Care and Use Committee (IACUC) of Kaohsiung Chang Gung Memorial Hospital.

### 2.2. RNA Isolation

Total RNA was extracted from the harvested nerve graft, whole blood, and serum by using the mirVana miRNA Isolation Kit (Ambion, Austin, TX, USA). For miRNA array, purified RNA yield was determined on the basis of the absorbance at 260 nm by using an SSP-3000 NanoDrop spectrophotometer (Infinigen Biotech, City of Industry, CA, USA), and RNA quality was evaluated with a Bioanalyzer 2100 system (Agilent Technologies, Palo Alto, CA, USA).

### 2.3. Small RNA Library Preparation

Small RNAs had linkers ligated to them and bar-coded cDNAs were prepared using the TruSeq Small RNA Sample Prep Kit (Illumina) following the manufacturer's instructions. Individual libraries were analyzed on a Bioanalyzer (Agilent) to detect the presence of linkered cDNA of the appropriate size (135–165 bp), and 11 bar-coded libraries were pooled into one sample by mixing 2 ng of the 135–165 bp peak from each sample, as determined by the Bioanalyzer. The 135–165-bp peak of the pooled cDNAs was purified from the mixed sample by using the Pippin Prep DNA Size Selection System (Sage Science) and confirmed by the Bioanalyzer.

### 2.4. Illumina Small RNA Deep Sequencing

Sequencing of the pooled libraries was performed in one lane of the Illumina HiSeq2000 Sequencer at the miRBase v19, and 50-bp single-end reads of the libraries were obtained. After indexing and trimming linker sequences, reads with a minimum length of 15 nt and with <3 terminal mismatches in the sequence were sorted and counted for the following analysis. The deep sequencing experiment was performed by BGI Tech Solutions Co., Ltd. (BGI Tech, Shenzhen, China).

### 2.5. Gene Ontology (GO) Enrichment and Kyoto Encyclopedia of Genes and Genomes (KEGG) Pathway Analyses

First, this method mapped all target gene candidates to GO terms in the database (http://www.geneontology.org/), calculated gene numbers for each term, and then used hypergeometric test to find significantly enriched GO terms in target gene candidates, compared to the reference gene background. The Bonferroni correction for *p* value was used to obtain a corrected *p* value. GO terms with a corrected *p* value of ≤ 0.05 were defined as significantly enriched in the target gene candidates. Pathway analysis from a major public pathway-related database KEGG [19] was performed to identify significantly enriched metabolic pathways or signal transduction pathways involving the target gene candidates, compared to the reference gene background. Genes with a false discovery rate of ≤0.05 were considered as significantly enriched in the target gene candidates.

## 3. Results

### 3.1. Sequence Analysis of Small RNAs

**Figure 1:**
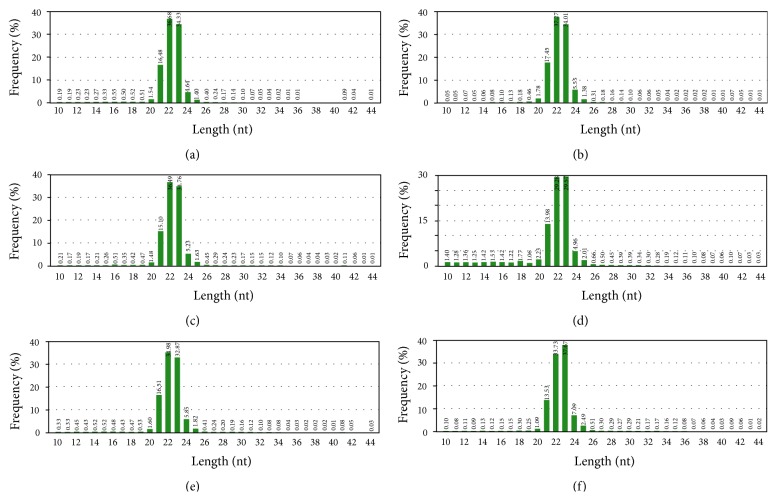
Length distribution and abundance of small RNA sequences as observed by Illumina small RNA deep sequencing in six libraries. Nerve allograft transplantation with FK506 immunosuppression at 3 d (a), 7 d (b), or 14 d (c). Nerve allograft transplantation without FK506 immunosuppression at 3 d (d), 7 d (e), or 14 d (f).

To profile the circulating miRNAs expressed during nerve allograft transplantation, six small RNA libraries were generated from 3-, 7-, and 14-day serum samples of mice receiving nerve allograft transplantation with or without FK506 immunosuppression (hereafter indicated as (F)3d, (F)7d, (F)14d, (N)3d, (N)7d, and (N)14d). The libraries were sequenced by Illumina small RNA deep sequencing technology. The number and proportion of the categories of small RNAs found are supplied in the Supplementary File (in Supplementary Material available online at http://dx.doi.org/10.1155/2015/863192). In total, 7785957, 5978171, 5978171, 5574644, 5988924, and 5975945 high-quality raw reads were obtained from the serum libraries of (F)3d, (F)7d, (F)14d, (N)3d, (N)7d, and (N)14d mice, respectively. After filtering the low-quality sequences, empty adaptors, and single-read sequences, 5458187 (70.1%), 5272112 (88.19%), 5670065 (94.85%), 5199001 (93.26%), 5710818 (95.36%), and 5334192 (89.26%) clean reads, 18–30 nt in length, were selected for further analysis from the serum libraries of (F)3d, (F)7d, (F)14d, (N)3d, (N)7d, and (N)14d mice, respectively (Supplementary File 1: Table S1). Among the selected reads, 2689173, 2853061, 2750572, 2615969, 3120416, and 2574471 sequences from the serum libraries of (F)3d, (F)7d, (F)14d, (N)3d, (N)7d, and (N)14d mice, respectively, mapped perfectly to the mouse genome, amounting to 44.14% and 49.94% of the total reads, respectively; and 2602557, 2763676, 2657576, 2590795, 3088195, and 2555335 reads in the serum libraries of (F)3d, (F)7d, (F)14d, (N)3d, (N)7d, and (N)14d mice, respectively, were found to be similar to miRNAs. The rest of the sequences were found to be other types of RNA, including noncoding RNA, rRNA, scRNA, snRNA, snoRNA, srpRNA, and tRNA. The size distribution of small RNAs (sRNAs) was similar in the two libraries, and majority of them ranged from 21 to 24 nt in length (Figure 1). The most abundant size class was 22 nt, which accounted for 36.68%, 37.27%, 36.49%, 29.28%, 34.98%, and 33.73% of the total reads in (F)3d, (F)7d, (F)14d, (N)3d, (N)7d, and (N)14d mice, respectively, followed by 23 nt (34.33%, 34.01%, 34.76%, 29.57%, 32.87%, and 37.67%, resp.).

### 3.2. Identification of Known miRNAs

The Illumina small RNA deep sequencing approach allows us to determine the relative abundance of various miRNA families by calculating the sequencing frequency. To investigate the expression of known miRNAs during nerve allograft transplantation, identified small RNA sequences were compared with known mature miRNAs in miRBase (version 19, 2012). The results showed that 507 mature miRNAs were present in these six libraries (Supplementary File 1: Table S2). Among them, there were 147 mature miRNAs with ≥50 sequence reads (Supplementary File 1: Table S3) and nine mature miRNAs with ≥5000 sequence reads in at least one of these six libraries (Supplementary File 1: Table S4). In these libraries, known miRNAs had a broad range of expression levels; some (such as miR-486-5p and miR-3107-5p) were found to have more than hundreds of thousands of sequence reads, while many others had ≤20, indicating that expression varies significantly among different miRNA families. The proportion of different categories of small RNAs often reflects the roles in a particular stage and the associated biological mechanisms during nerve graft allotransplantation. With ≥5,000 reads, the following four miRNAs were dominantly expressed in all these six libraries: miR-25-3p, miR-486-5p, miR-3107-5p, and miR-92a-3p (Supplementary File 1: Table S5). In addition to miR-486-5p and miR-3107-5p, which have ≥200000 sequence reads in all six libraries, miR-92a-3p and miR-25-3p were the third and fourth most abundant miRNA in (F)7d (22068 and 13644 reads, resp.), (F)14d (19175 and 15427 reads, resp.), (N)3d (18312 and 10353 reads, resp.), and (N)14d (19852 and 8768 reads, resp.) samples (Supplementary File 1: Table S6).

### 3.3. Differentially Expressed miRNAs

**Figure 2:**
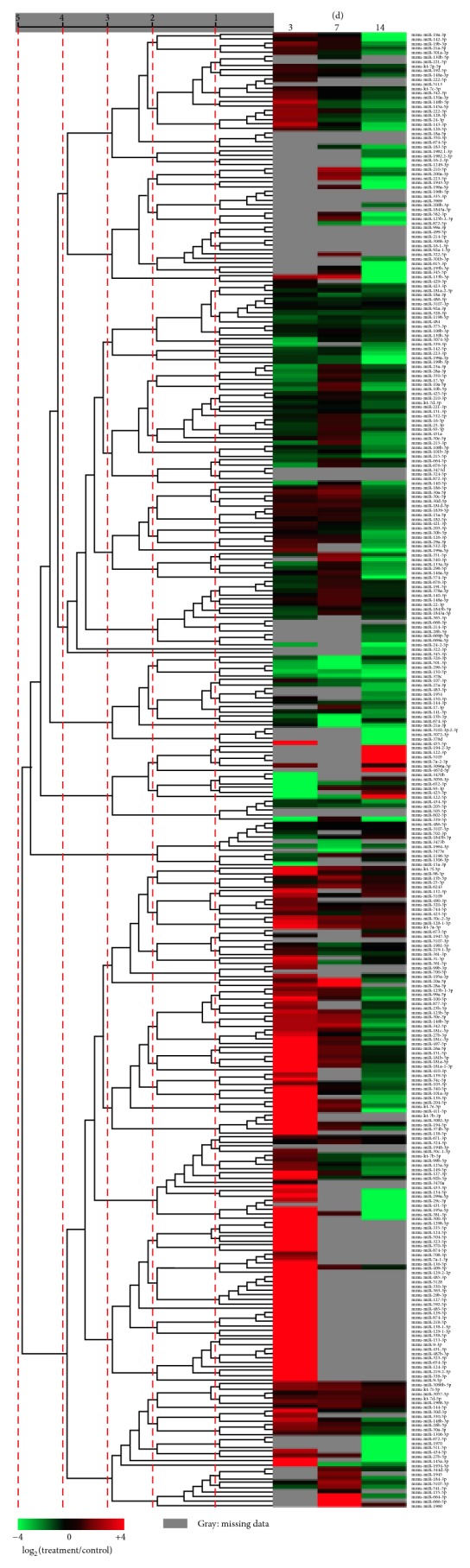
Unsupervised hierarchical clustering of miRNA expression. Hierarchical clustering of miRNA differentially expressed in the sera of the mice receiving nerve allotransplantation with or without daily injection of 1 mg/(kg·d) FK506 for immunosuppression.

**Figure 3:**
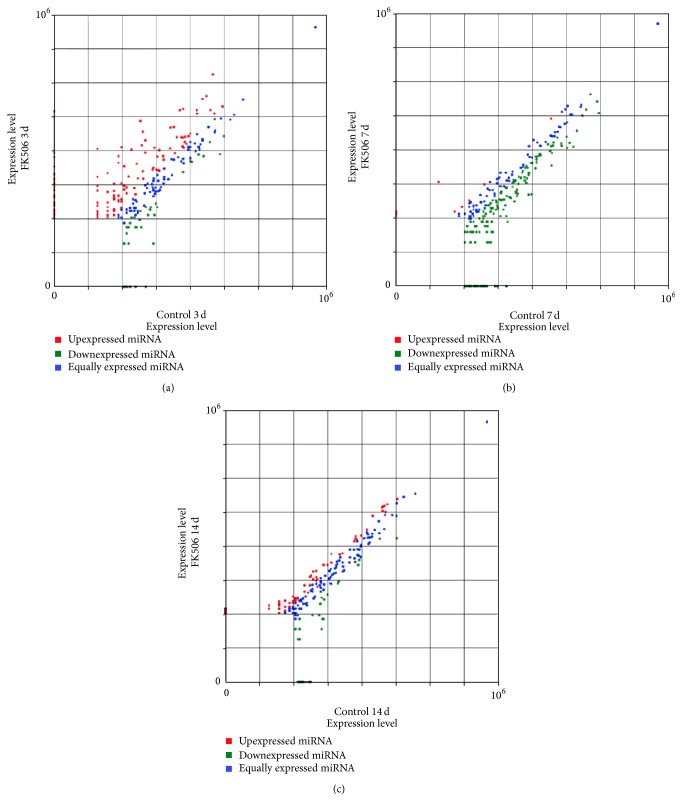
Comparison of miRNA expression levels at 3, 7, and 14 d in the sera of the mice receiving nerve allotransplantation with or without daily injection of 1 mg/(kg·d) FK506 for immunosuppression. Red dots: upregulated miRNAs, green dots: downregulated miRNAs, and blue dots: equally expressed miRNAs.

**Figure 4:**
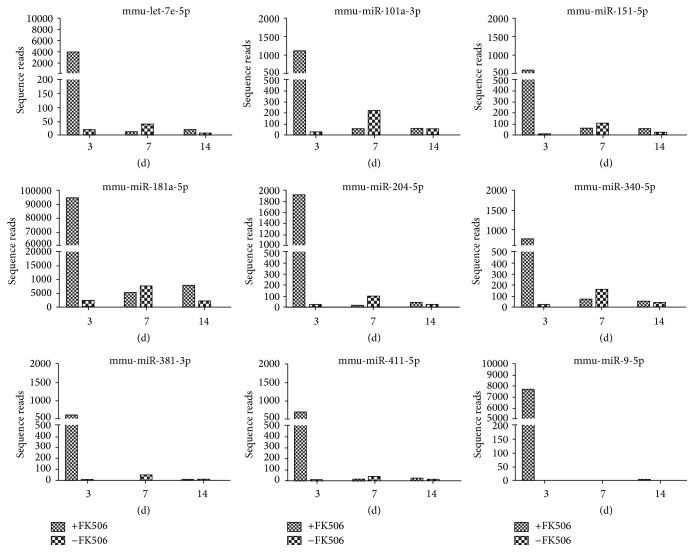
Sequence reads of the nine upregulated circulating miRNAs throughout the experimental period in the sera of the mice receiving nerve allotransplantation with or without daily injection of 1 mg/(kg·d) FK506 for immunosuppression.

According to the changes in relative miRNA abundance between the two serum libraries obtained from mice receiving nerve allograft transplantation with and without FK506 immunosuppression at the same indicated time points (3, 7, and 14 d), unsupervised hierarchical clustering (Figure 2) as well as supervised comparison of all significantly differentially expressed miRNAs was conducted to categorize the samples from experimental subjects at different days into different groups by using univariate analyses (*p* < 0.001).  Figure 3 illustrates the differentially expressed circulating miRNAs at different times between mice receiving allotransplantation with and without FK506 immunosuppression. The differentially expressed miRNAs with ≥500 sequence reads in at least one of the libraries were selected for further comparison, which identified nine miRNAs with differences greater than 5-fold between the two libraries at 3 d (Table 1), but none was identified at 7 and 14 d, respectively. Among the nine upregulated miRNAs (let-7e-5p, miR-101a-3p, miR-151-5p, miR-181a-5p, miR-204-5p, miR-340-5p, miR-381-3p, miR-411-5p, miR-9-5p, and miR-219-2-3p), miR-9-5p had the highest fold-change (≥50-fold at 3 d), followed by miR-340-5p with 38.8-fold. Therefore, we focused on the differentially expressed miRNAs at 3 d with ≥500 sequence reads and studied their expression profile throughout the course of the experiment. Nine candidate miRNAs (let-7e-5p, miR-101a-3p, miR-151-5p, miR-181a-5p, miR-204-5p, miR-340-5p, miR-381-3p, miR-411-5p, and miR-9-5p) were identified. Although all these nine miRNAs had high-to-very-high sequence reads (576–9474) in the mice undergoing allotransplantation with FK506 immunosuppression at 3 d, compared to that observed in those without immunosuppression, none of these miRNAs showed high expression at 7 d or 14 d (Figure 4). These results indicate that different miRNAs have clearly different specific expression levels at different times.

### 3.4. Target Prediction and Functional Annotation

To further understand the physiological functions and biological processes involving the nine upregulated miRNAs at 3 d in the sera of mice receiving nerve allotransplantation with FK506 treatment, target prediction was performed by integrating five common public databases: miRanda (http://www.microrna.org/microrna/home.do), MirTarget2 (http://mirdb.org/miRDB/), PicTar (http://pictar.mdc-berlin.de/), PITA (http://genie.weizmann.ac.il/index.html), and RNAhybrid (http://bibiserv.techfak.uni-bielefeld.de/rnahybrid/). Only the gene with miRNA target interactions predicted by at least three of these five target prediction programs was chosen (Supplementary File 2). GO enrichment and KEGG pathway analyses were performed to identify the functional modules regulated by these nine miRNAs. The results of GO annotation enrichment analysis regarding biological processes, cellular components, and molecular functions are listed in Supplementary File 3. KEGG pathway annotation showed that 21,703 background genes were annotated for 270 biological functions. KEGG pathway analysis revealed 39 pathways that were significantly enriched with these miRNA targets (Supplementary File 4). Among them, the MAPK signaling pathway, Wnt signaling pathway, pathways involved in cancer, focal adhesion, gap junction formation, and degradation, and axon guidance, calcium signaling pathway, and Notch signaling pathway were ranked among the most enriched pathways. These results suggested that, during early stages, these targets show a high possibility of being regulated by miRNAs in cases of nerve allotransplantation with FK506 immunosuppression, although the possibility of false-positive prediction from the prediction algorithm always exists.

## 4. Discussion

The Illumina deep sequencing platform is efficient for miRNA discovery and is widely used to generate small RNA profiles in various organisms. In this study, we identified the circulating miRNAs in a mouse model of nerve allograft transplantation under FK506 immunosuppression by Illumina small RNA deep sequencing. The sequence analysis showed that the dominant size of small RNAs in circulation after nerve allotransplantation in the presence or absence of FK506 immunosuppression was 22 nt, followed by 23 nt. This result is typical of Dicer-processed small RNAs and was consistent with the known size of ~22 nt for miRNAs. Selection of the differentially expressed miRNAs under the relatively strict conditions (≥500 sequence reads in at least one of the libraries selected for comparison, ≥5-fold difference in expression, and a *p* value of ≤ 0.01) identified nine upregulated miRNAs (let-7e-5p, miR-101a-3p, miR-151-5p, miR-181a-5p, miR-204-5p, miR-340-5p, miR-381-3p, miR-411-5p, miR-9-5p, and miR-219-2-3p) at 3 d, but none at 7 d or 14 d, suggesting that these upregulated miRNAs impact biological functions, particularly during the early stages after nerve allotransplantation with FK506 immunosuppression.

Among these nine upregulated circulating miRNAs, miR-9-5p had the highest fold-change of ≥50-fold, followed by miR-340-5p with 38.8-fold. Correlation of miR-9-3p with the genes involved in immune/inflammatory responses (e.g., IFNG and IL17F), apoptosis (e.g., PDCD4 and PTEN), and cell proliferation (e.g., NKX3-1 and GADD45A) has been reported in stimulated human peripheral blood lymphocytes [20]. miR-340-5p is confirmed to be related to nerve injury and elevated at all five time points, from 1 h to 7 d, after traumatic brain injury [21]. Further, none of the remaining seven upregulated miRNAs has been explored or validated, in available literature, to be related to immune function, nerve injury, or nerve regeneration. In this study, KEGG pathway analysis of these miRNA targets revealed that MAPK signaling pathway, Wnt signaling pathway, pathways involved in cancer, focal adhesion, gap junction formation and degradation, and axon guidance, calcium signaling pathway, and Notch signaling pathway are ranked among the most enriched pathways. Although most of these pathways have been reported to be involved in the processes during nerve injury or nerve regeneration, these pathways are quite heterogeneous, and their role in nerve allotransplantation has seldom been explored. However, it is important to identify the origin and target cells of these circulating miRNAs prior to the discussion of these pathways. Whether these circulating miRNAs have originated from T cells, blood cells, or endothelial cells of the circulating system is unknown, and their target cells are yet to be identified. Unfortunately, at present, there is no standard protocol to identify the origin of circulating miRNAs. Notably, although the origin of circulating miRNAs is debatable, the expression profile of circulating miRNAs is obviously different from that of the miRNAs in pathological tissues [11, 22]. The immunosuppressive drug FK506 has been proved to exert neuroprotective and neurotrophic actions in experimental models; it increases neurite elongation and accelerates the rate of nerve regeneration in vitro and in vivo [7, 23]. In this study, miRNAs are differentially expressed between mice receiving allogeneic grafts with and without immunosuppression during the early stages, suggesting that their important regulatory function was not the suppression of graft rejection, a phenomenon that generally occurs at 7 d after nerve allotransplantation in the absence of immunosuppression. Whether these circulating miRNAs play a potential role in the nerve regeneration-enhancing activity of FK506 requires further investigation.

Although allograft biopsy is the gold standard for diagnosis of conditions such as acute rejection, disease recurrence, and drug toxicity [24, 25], it is impractical to acquire a nerve biopsy in clinical settings. Circulating miRNAs, owing to the noninvasive nature of their detection, their disease specificity, and the availability of accurate techniques for detection and monitoring, may be developed as excellent biomarkers of allograft injury and function [26]. For example, it had been reported that circulating cell-free miRNAs correlate with specific hepatic injury and thus may serve as feasible monitoring and outcome-predictive biomarkers in liver transplantation [27]. Differential expression of miRNAs was also observed in urine samples between patient groups with chronic allograft dysfunction [28]. Therefore, miRNAs may serve as potential biomarkers for organ quality, ischemia-reperfusion injury, acute rejection, tolerance, and chronic allograft dysfunction, emphasizing their mechanistic and clinical applications [26, 29]. In this study, our additional goal was to identify possible upregulated circulating miRNAs after nerve allotransplantation in mice with or without immunosuppression and then determine whether these identified circulating miRNAs may be used as targets for monitoring or therapeutic intervention. However, when compared to the miRNAs found in mice with untreated isograft, no circulating miRNA with significantly increased expression was found at 7 d or 14 d in both groups. The presence of these nine upregulated circulating miRNAs only at 3 d, but not at 7 d or 14 d, after nerve allotransplantation with FK506 immunosuppression, indicates that these upregulated miRNAs are not ideal biomarkers for monitoring the status of deep-buried nerve allografts. However, the underlying mechanistic basis for the expression of circulating miRNA during the early stages of nerve allotransplantation under immunosuppression, but not under allogeneic condition, remains an area of utmost interest. In our prior report that profiles the circulating miRNAs expression in a mouse model of nerve allotransplantation, we used BALB/c mice as recipient animals and C57BL/6 mice as donor animals for full major histocompatibility complex (MHC) disparity [30, 31] and identified the circulating miR-320, miR-762, and miR-423-5p as potential biomarkers for monitoring the immunosuppression status of the nerve allograft [18]. We also found that the expression of all these 3 upregulated circulating miRNAs significantly decreased at 2, 4, and 6 d after discontinuation of FK506 immunosuppression [18]. In contrast, in this study that used C57BL/6 mice as recipient animals and BALB/c mice as donor animals, no ideal upregulated miRNAs are identified for monitoring the status of deep-buried nerve allografts. The results indicate that a distinct expression of circulating miRNAs may exist even between different kinds of the same species and also suggest the difficulty to translate the research results of mice to the human being.

In conclusion, this study identified nine upregulated circulating miRNAs after nerve allotransplantation with FK506 immunosuppression by Illumina small RNA deep sequencing. These miRNAs affect biological functions, in particular during the early stages after nerve allotransplantation under immunosuppression.

## Supplementary Material

Expression profile of the miRNAs by Illumina small RNA deep sequencing.

## Figures and Tables

**Table 1:** Differentially expressed miRNAs with ≥500 sequence reads and regulated ≥5-fold in the sera of C57BL/6 mice receiving nerve allotransplantation with or without immunosuppression for 3 d^*∗∗*^, *p* value < 0.01.

miR name	Fold-change	*p* value
mmu-let-7e-5p	7.7	0^*∗∗*^
mmu-miR-101a-3p	5.4	4.4*E* − 274^*∗∗*^
mmu-miR-151-5p	5.8	4.1*E* − 150^*∗∗*^
mmu-miR-181a-5p	5.2	0^*∗∗*^
mmu-miR-204-5p	6.5	0^*∗∗*^
mmu-miR-340-5p	38.8	8.3*E* − 32^*∗∗*^
mmu-miR-381-3p	9.2	8.6*E* − 176^*∗∗*^
mmu-miR-411-5p	7.0	4.7*E* − 186^*∗∗*^
mmu-miR-9-5p	>50	8.3*E* − 32^*∗∗*^
