# Supplementary figures and images for: Identification of Circulating miRNAs in a Mouse Model of Nerve Allograft Transplantation under FK506 Immunosuppression by Illumina Small RNA Deep Sequencing
Source: Dis Markers. 2015 Sep 8;2015:863192. doi: 10.1155/2015/863192 (PMC4578739; doi:10.1155/2015/863192)

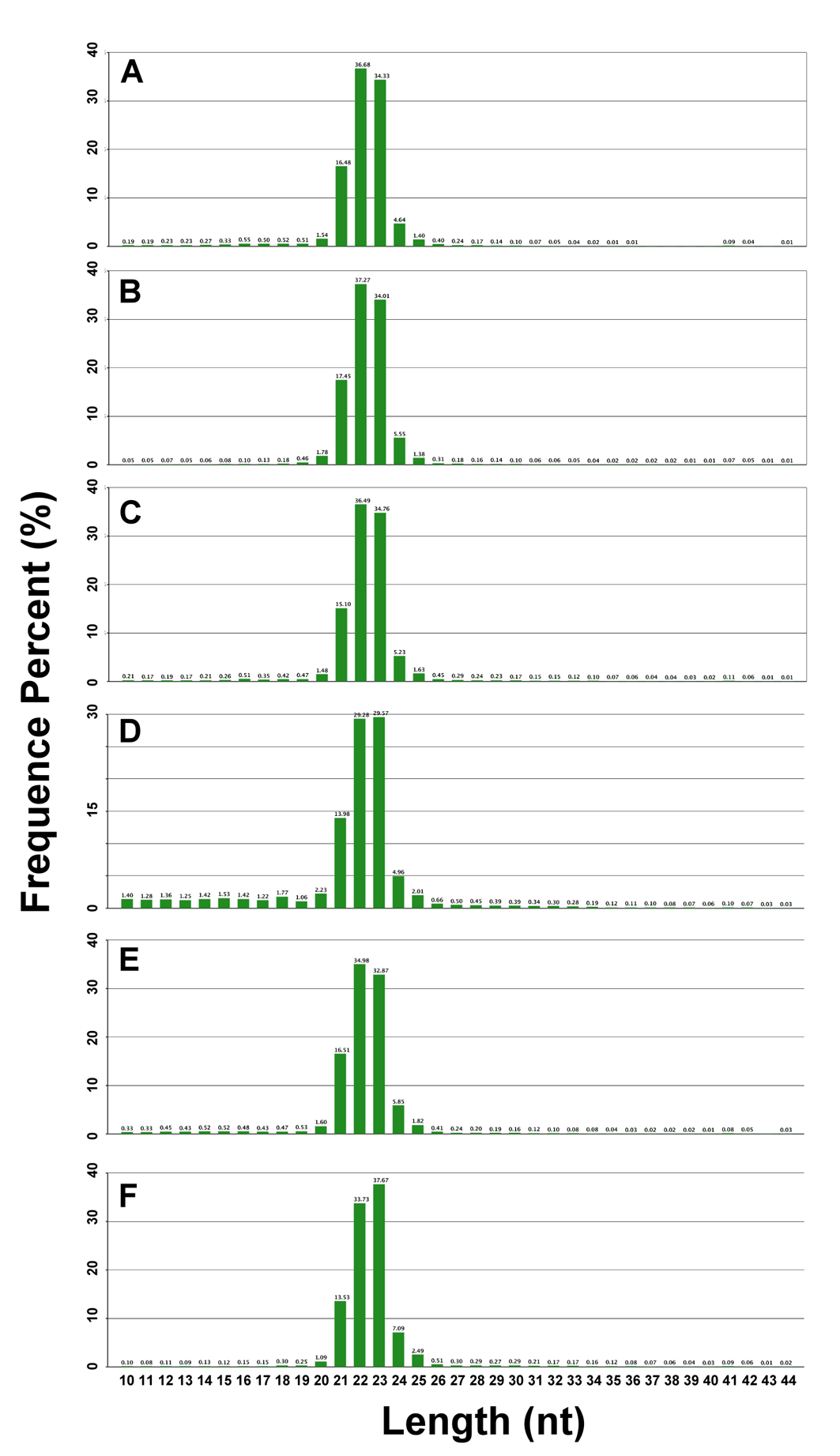

Supplement: Supplementary file 1 — Expression profile of the miRNAs by Illumina small RNA deep sequencing. [file 863192.f1.zip › 863192.f1/Figure 1.tif]

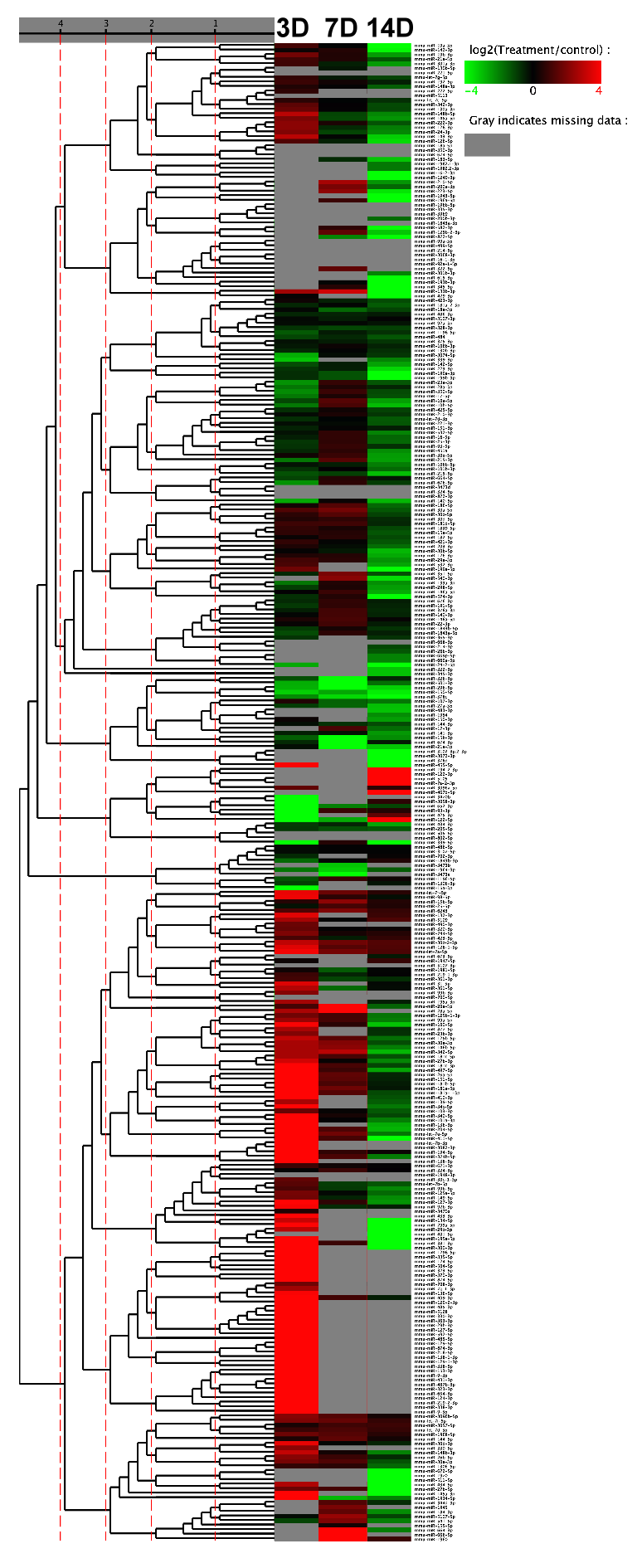

Supplement: Supplementary file 1 — Expression profile of the miRNAs by Illumina small RNA deep sequencing. [file 863192.f1.zip › 863192.f1/Figure 2.tif]

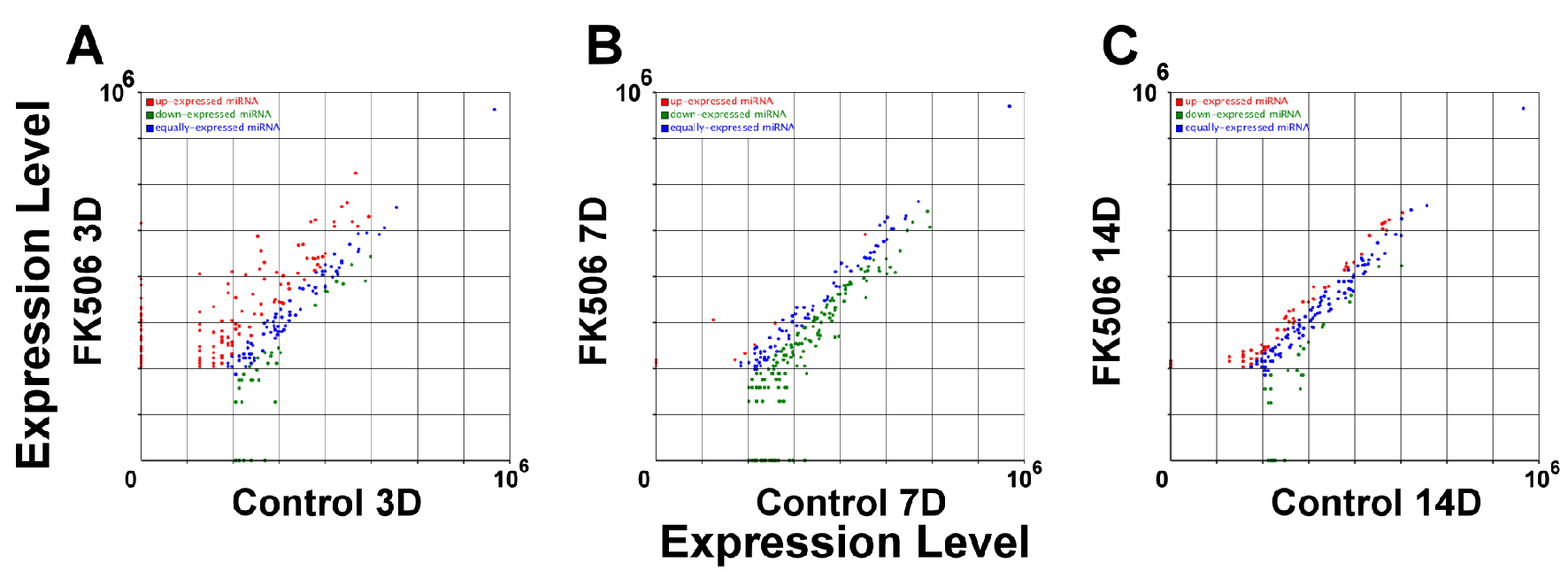

Supplement: Supplementary file 1 — Expression profile of the miRNAs by Illumina small RNA deep sequencing. [file 863192.f1.zip › 863192.f1/Figure 3.tif]

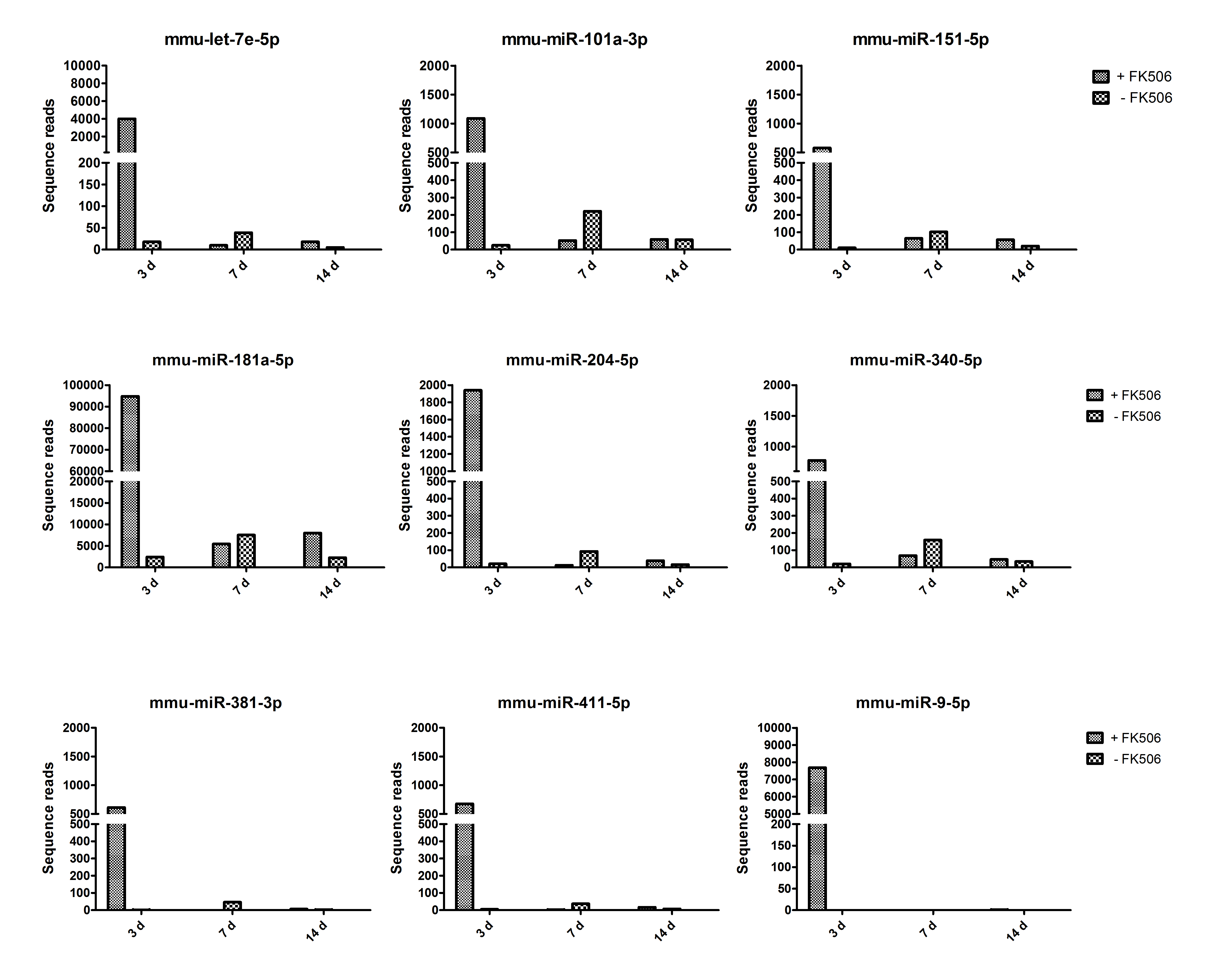

Supplement: Supplementary file 1 — Expression profile of the miRNAs by Illumina small RNA deep sequencing. [file 863192.f1.zip › 863192.f1/Figure 4.tif]
